# 11*H*-Dibenzo[*b*,*e*]azepine-6-carbonitrile

**DOI:** 10.1107/S1600536808033072

**Published:** 2008-10-18

**Authors:** Guo-Dong Fan, Yu-Liang Xiao, Gui-Rong You, Gui-Yun Duan

**Affiliations:** a College of Chemistry and Chemical Engineering, Shaanxi University of Science and Technology, Xi’an 710021, People’s Republic of China; b College of Pharmaceutical Sciences, Taishan Medical College, Tai’an 271016, People’s Republic of China

## Abstract

The title compound, C_15_H_10_N_2_, crystallizes with two independent mol­ecules in the asymmetric unit. The two benzene rings make dihedral angles of 60.32 (2) and 61.35 (3)°. The crystal packing is stabilized by weak π–π stacking inter­actions [centroid-to-centroid distances = 3.673 (4) and 3.793 (4) Å].

## Related literature

For discussions of the biological activity of the title compound, see: Bakker *et al.* (2000 ▶); Bielory & Ghafoor (2005 ▶); Schmutz *et al.* (1967 ▶). For a similar structure, see: Li *et al.* (2006 ▶).
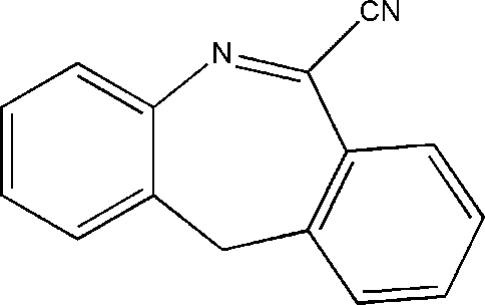


## Experimental

### 

#### Crystal data

C_15_H_10_N_2_
*M* *_r_* = 218.25
Triclinic, 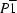

*a* = 10.125 (2) Å
*b* = 10.275 (2) Å
*c* = 12.749 (3) Å
α = 105.96 (3)°
β = 99.18 (2)°
γ = 109.04 (3)°
*V* = 1159.2 (6) Å^3^
*Z* = 4
Mo *K*α radiation
μ = 0.08 mm^−1^
*T* = 273 (2) K
0.15 × 0.12 × 0.10 mm

#### Data collection

Bruker SMART CCD area-detector diffractometer
Absorption correction: multi-scan (*SADABS*; Sheldrick, 1996 ▶) *T*_min_ = 0.989, *T*_max_ = 0.993
12026 measured reflections
4084 independent reflections
3382 reflections with *I* > 2σ(*I*)
*R*_int_ = 0.021

#### Refinement

*R*[*F* ^2^ > 2σ(*F* ^2^)] = 0.036
*wR*(*F* ^2^) = 0.097
*S* = 1.03
4084 reflections
308 parameters
H-atom parameters constrained
Δρ_max_ = 0.19 e Å^−3^
Δρ_min_ = −0.13 e Å^−3^

### 

Data collection: *SMART* (Bruker, 2001 ▶); cell refinement: *SAINT* (Bruker, 2001 ▶); data reduction: *SAINT*; program(s) used to solve structure: *SHELXTL* (Sheldrick, 2008 ▶); program(s) used to refine structure: *SHELXTL*; molecular graphics: *SHELXTL*; software used to prepare material for publication: *SHELXTL*.

## Supplementary Material

Crystal structure: contains datablocks I, global. DOI: 10.1107/S1600536808033072/sg2271sup1.cif

Structure factors: contains datablocks I. DOI: 10.1107/S1600536808033072/sg2271Isup2.hkl

Additional supplementary materials:  crystallographic information; 3D view; checkCIF report

## Figures and Tables

**Table 1:** Selected interatomic distances (Å) *Cg*1 is the centroid of the ring C8–C13 and *Cg*2 is the centroid of the ring C23–C28.

*Cg*1⋯*Cg*1^i^	3.673 (4)
*Cg*2⋯*Cg*2^ii^	3.793 (4)

Symmetry codes: (i) 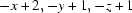; (ii) 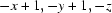.

**Fig. 1.**
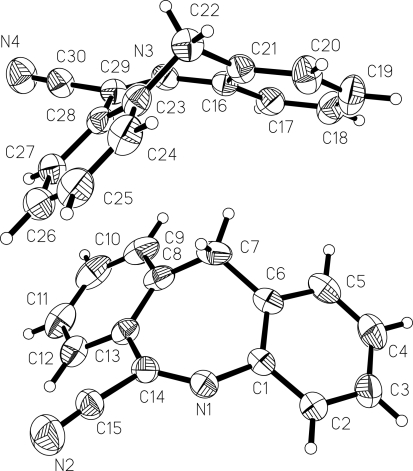
View of the title compound (I), with displacement ellipsoids drawn at the 35% probability level.

## supplementary crystallographic information

### Comment

The title compound, (1), is an intermediate in the synthesis of Epinastine which is an antihistamine agent (Bakker *et al.*, 2000; Bielory & Ghafoor, 2005), and was first synthesized in 1967 (Schmutz *et al.*, 1967).

Compound (1) crystallizes with two independent molecules in the asymmetric unit (Fig. 1), all bond lengths and angles are normal and in a good agreement with those reported previously (Li *et al.*, 2006). The dihedral angles between the planes of benzene rings in the two independent molecules are 60.32 (2) and 61.35 (3)°. π–π stacking interactions (Table 1) are present in the structure (*Cg*1: C8–C13; *Cg*2: C23–C28).

### Experimental

Compound (1) was synthesized from 6-chlor-11*H*-dibenzo[b,e]azepine (1 mmol, 0.23 g) and sodium cyanade (1.1 mmol, 0.05 g) in 10 ml DMSO as solvent at 363 K for 5 h to afford the title compound (Yield 73%, 0.16 g). Crystals suitable for X-ray diffraction analysis were obtained by slow evaporation of a methanol solution at room temperature for one week.

### Refinement

All H atoms were placed in calculated positions, with C—H = 0.93 or 0.97 Å, and included in the final cycles of refinement using a riding model, with *U*_iso_(H) = 1.2 times *U*_eq_(C).

### Crystal data

**Table:**

C_15_H_10_N_2_	*Z* = 4
*M**_r_* = 218.25	*F*(000) = 456
Triclinic, *P*1	*D*_x_ = 1.251 Mg m^−^^3^
Hall symbol: -P 1	Mo *K*α radiation, λ = 0.71073 Å
*a* = 10.125 (2) Å	Cell parameters from 4851 reflections
*b* = 10.275 (2) Å	θ = 2.2–28.0°
*c* = 12.749 (3) Å	µ = 0.08 mm^−^^1^
α = 105.96 (3)°	*T* = 273 K
β = 99.18 (2)°	Block, brown
γ = 109.04 (3)°	0.15 × 0.12 × 0.10 mm
*V* = 1159.2 (6) Å^3^	

### Data collection

**Table:**

Bruker SMART CCD area-detector diffractometer	4084 independent reflections
Radiation source: fine-focus sealed tube	3382 reflections with *I* > 2σ(*I*)
graphite	*R*_int_ = 0.021
φ and ω scans	θ_max_ = 25.0°, θ_min_ = 2.2°
Absorption correction: multi-scan (SADABS; Sheldrick, 1996)	*h* = −12→12
*T*_min_ = 0.989, *T*_max_ = 0.993	*k* = −12→12
12026 measured reflections	*l* = −14→15

### Refinement

**Table:**

Refinement on *F*^2^	Secondary atom site location: difference Fourier map
Least-squares matrix: full	Hydrogen site location: inferred from neighbouring sites
*R*[*F*^2^ > 2σ(*F*^2^)] = 0.036	H-atom parameters constrained
*wR*(*F*^2^) = 0.097	*w* = 1/[σ^2^(*F*_o_^2^) + (0.0446*P*)^2^ + 0.1613*P*] where *P* = (*F*_o_^2^ + 2*F*_c_^2^)/3
*S* = 1.03	(Δ/σ)_max_ < 0.001
4084 reflections	Δρ_max_ = 0.19 e Å^−^^3^
308 parameters	Δρ_min_ = −0.13 e Å^−^^3^
0 restraints	Extinction correction: SHELXTL (Sheldrick, 2008), Fc^*^=kFc[1+0.001xFc^2^λ^3^/sin(2θ)]^-1/4^
Primary atom site location: structure-invariant direct methods	Extinction coefficient: 0.037 (2)

### Special details

**Table:**

Geometry. All e.s.d.'s (except the e.s.d. in the dihedral angle between two l.s. planes) are estimated using the full covariance matrix. The cell e.s.d.'s are taken into account individually in the estimation of e.s.d.'s in distances, angles and torsion angles; correlations between e.s.d.'s in cell parameters are only used when they are defined by crystal symmetry. An approximate (isotropic) treatment of cell e.s.d.'s is used for estimating e.s.d.'s involving l.s. planes.
Refinement. Refinement of *F*^2^ against ALL reflections. The weighted *R*-factor *wR* and goodness of fit *S* are based on *F*^2^, conventional *R*-factors *R* are based on *F*, with *F* set to zero for negative *F*^2^. The threshold expression of *F*^2^ > σ(*F*^2^) is used only for calculating *R*-factors(gt) *etc*. and is not relevant to the choice of reflections for refinement. *R*-factors based on *F*^2^ are statistically about twice as large as those based on *F*, and *R*- factors based on ALL data will be even larger.

### Fractional atomic coordinates and isotropic or equivalent isotropic displacement parameters (Å^2^)

**Table:**

	*x*	*y*	*z*	*U*_iso_*/*U*_eq_	
N1	0.93668 (11)	0.20617 (11)	0.18911 (9)	0.0486 (3)	
N2	1.10390 (18)	0.47578 (17)	0.10411 (14)	0.0874 (4)	
N3	0.33399 (10)	0.33411 (11)	0.30617 (9)	0.0474 (3)	
N4	0.42697 (16)	0.69062 (15)	0.35893 (13)	0.0816 (4)	
C1	0.87147 (12)	0.11423 (13)	0.24622 (10)	0.0466 (3)	
C2	0.93247 (14)	0.01429 (14)	0.25883 (12)	0.0568 (3)	
H2A	1.0096	0.0105	0.2288	0.068*	
C3	0.87991 (17)	−0.07883 (16)	0.31523 (14)	0.0699 (4)	
H3B	0.9238	−0.1425	0.3261	0.084*	
C4	0.76259 (18)	−0.07763 (18)	0.35547 (14)	0.0747 (5)	
H4A	0.7275	−0.1399	0.3944	0.090*	
C5	0.69679 (16)	0.01499 (17)	0.33859 (13)	0.0679 (4)	
H5A	0.6151	0.0122	0.3640	0.082*	
C6	0.74956 (13)	0.11314 (15)	0.28418 (11)	0.0532 (3)	
C7	0.67936 (14)	0.21544 (17)	0.26432 (13)	0.0644 (4)	
H7A	0.6553	0.2013	0.1841	0.077*	
H7B	0.5900	0.1936	0.2871	0.077*	
C8	0.78013 (14)	0.37191 (16)	0.33094 (11)	0.0549 (3)	
C9	0.74955 (18)	0.4600 (2)	0.41954 (13)	0.0707 (4)	
H9A	0.6653	0.4210	0.4403	0.085*	
C10	0.8421 (2)	0.6044 (2)	0.47717 (14)	0.0800 (5)	
H10A	0.8204	0.6615	0.5369	0.096*	
C11	0.9659 (2)	0.66483 (19)	0.44740 (13)	0.0732 (4)	
H11A	1.0271	0.7630	0.4859	0.088*	
C12	0.99953 (16)	0.58006 (15)	0.36048 (12)	0.0596 (4)	
H12A	1.0832	0.6213	0.3398	0.071*	
C13	0.90890 (13)	0.43261 (14)	0.30305 (10)	0.0486 (3)	
C14	0.95118 (13)	0.34087 (14)	0.21488 (10)	0.0469 (3)	
C15	1.03427 (15)	0.41783 (15)	0.14911 (12)	0.0549 (3)	
C16	0.30864 (12)	0.18357 (13)	0.26934 (11)	0.0457 (3)	
C17	0.32200 (14)	0.12481 (16)	0.35496 (12)	0.0570 (3)	
H17A	0.3485	0.1854	0.4305	0.068*	
C18	0.29646 (18)	−0.02147 (18)	0.32903 (16)	0.0717 (4)	
H18A	0.3087	−0.0591	0.3867	0.086*	
C19	0.2528 (2)	−0.11204 (18)	0.21765 (17)	0.0804 (5)	
H19A	0.2344	−0.2115	0.1997	0.097*	
C20	0.23630 (18)	−0.05569 (16)	0.13243 (15)	0.0703 (4)	
H20A	0.2060	−0.1184	0.0573	0.084*	
C21	0.26371 (13)	0.09205 (14)	0.15582 (11)	0.0508 (3)	
C22	0.24469 (15)	0.15472 (15)	0.06392 (11)	0.0577 (4)	
H22A	0.1773	0.2029	0.0739	0.069*	
H22B	0.2046	0.0762	−0.0095	0.069*	
C23	0.38852 (15)	0.26340 (15)	0.06805 (11)	0.0518 (3)	
C24	0.45555 (19)	0.23862 (18)	−0.01782 (13)	0.0684 (4)	
H24A	0.4114	0.1515	−0.0800	0.082*	
C25	0.5865 (2)	0.3412 (2)	−0.01221 (15)	0.0777 (5)	
H25A	0.6302	0.3222	−0.0704	0.093*	
C26	0.65358 (17)	0.4714 (2)	0.07818 (15)	0.0715 (4)	
H26A	0.7420	0.5402	0.0811	0.086*	
C27	0.58889 (15)	0.49905 (16)	0.16430 (13)	0.0583 (4)	
H27A	0.6326	0.5879	0.2249	0.070*	
C28	0.45808 (13)	0.39437 (14)	0.16093 (11)	0.0467 (3)	
C29	0.39603 (13)	0.42144 (13)	0.25784 (10)	0.0459 (3)	
C30	0.41483 (15)	0.57366 (16)	0.31439 (12)	0.0566 (3)	

### Atomic displacement parameters (Å^2^)

**Table:**

	*U*^11^	*U*^22^	*U*^33^	*U*^12^	*U*^13^	*U*^23^
N1	0.0475 (6)	0.0505 (6)	0.0503 (6)	0.0185 (5)	0.0175 (5)	0.0198 (5)
N2	0.0998 (11)	0.0880 (10)	0.0966 (11)	0.0383 (9)	0.0484 (9)	0.0511 (9)
N3	0.0454 (5)	0.0503 (6)	0.0456 (6)	0.0166 (5)	0.0171 (5)	0.0152 (5)
N4	0.0931 (10)	0.0557 (8)	0.0875 (10)	0.0253 (7)	0.0296 (8)	0.0140 (7)
C1	0.0414 (6)	0.0468 (7)	0.0461 (7)	0.0105 (5)	0.0116 (5)	0.0163 (6)
C2	0.0511 (7)	0.0501 (8)	0.0691 (9)	0.0171 (6)	0.0189 (6)	0.0229 (7)
C3	0.0716 (9)	0.0565 (9)	0.0850 (11)	0.0206 (7)	0.0198 (8)	0.0363 (8)
C4	0.0769 (10)	0.0655 (10)	0.0781 (11)	0.0102 (8)	0.0259 (9)	0.0385 (9)
C5	0.0525 (8)	0.0725 (10)	0.0725 (10)	0.0095 (7)	0.0263 (7)	0.0289 (8)
C6	0.0393 (6)	0.0592 (8)	0.0535 (8)	0.0112 (6)	0.0119 (6)	0.0184 (6)
C7	0.0404 (7)	0.0874 (11)	0.0719 (10)	0.0274 (7)	0.0195 (7)	0.0325 (8)
C8	0.0533 (7)	0.0761 (9)	0.0526 (8)	0.0391 (7)	0.0175 (6)	0.0299 (7)
C9	0.0765 (10)	0.1060 (14)	0.0618 (9)	0.0620 (10)	0.0305 (8)	0.0389 (10)
C10	0.1131 (14)	0.1013 (14)	0.0517 (9)	0.0765 (12)	0.0237 (9)	0.0231 (9)
C11	0.1017 (13)	0.0676 (10)	0.0545 (9)	0.0487 (9)	0.0093 (9)	0.0153 (8)
C12	0.0703 (9)	0.0566 (8)	0.0564 (8)	0.0307 (7)	0.0122 (7)	0.0224 (7)
C13	0.0523 (7)	0.0573 (8)	0.0453 (7)	0.0300 (6)	0.0120 (6)	0.0219 (6)
C14	0.0445 (6)	0.0529 (8)	0.0474 (7)	0.0200 (6)	0.0141 (5)	0.0220 (6)
C15	0.0579 (8)	0.0502 (8)	0.0647 (9)	0.0220 (6)	0.0287 (7)	0.0244 (7)
C16	0.0411 (6)	0.0497 (7)	0.0527 (8)	0.0192 (5)	0.0220 (5)	0.0205 (6)
C17	0.0569 (8)	0.0671 (9)	0.0591 (8)	0.0262 (7)	0.0290 (6)	0.0303 (7)
C18	0.0836 (11)	0.0774 (11)	0.0891 (12)	0.0452 (9)	0.0474 (9)	0.0513 (10)
C19	0.1060 (13)	0.0592 (9)	0.1064 (15)	0.0445 (9)	0.0607 (11)	0.0415 (10)
C20	0.0872 (11)	0.0526 (8)	0.0744 (10)	0.0277 (8)	0.0401 (9)	0.0163 (8)
C21	0.0508 (7)	0.0489 (7)	0.0543 (8)	0.0194 (6)	0.0234 (6)	0.0155 (6)
C22	0.0639 (8)	0.0562 (8)	0.0450 (7)	0.0212 (7)	0.0119 (6)	0.0103 (6)
C23	0.0657 (8)	0.0585 (8)	0.0451 (7)	0.0332 (7)	0.0212 (6)	0.0246 (6)
C24	0.0968 (11)	0.0771 (10)	0.0546 (9)	0.0472 (9)	0.0390 (8)	0.0314 (8)
C25	0.1023 (13)	0.1026 (14)	0.0778 (12)	0.0643 (11)	0.0602 (10)	0.0559 (11)
C26	0.0680 (9)	0.0919 (12)	0.0898 (12)	0.0400 (9)	0.0441 (9)	0.0609 (11)
C27	0.0586 (8)	0.0636 (9)	0.0660 (9)	0.0257 (7)	0.0236 (7)	0.0365 (7)
C28	0.0509 (7)	0.0541 (7)	0.0481 (7)	0.0268 (6)	0.0193 (6)	0.0262 (6)
C29	0.0439 (6)	0.0474 (7)	0.0453 (7)	0.0183 (5)	0.0119 (5)	0.0144 (6)
C30	0.0591 (8)	0.0516 (8)	0.0559 (8)	0.0182 (6)	0.0200 (6)	0.0156 (7)

### Geometric parameters (Å, °)

**Table:**

N1—C14	1.2833 (16)	C12—H12A	0.9300
N1—C1	1.4082 (16)	C13—C14	1.4728 (18)
N2—C15	1.1062 (17)	C14—C15	1.4805 (19)
N3—C29	1.2804 (16)	C16—C17	1.3940 (19)
N3—C16	1.4077 (16)	C16—C21	1.3956 (19)
N4—C30	1.1348 (18)	C17—C18	1.372 (2)
C1—C2	1.3909 (19)	C17—H17A	0.9300
C1—C6	1.3936 (18)	C18—C19	1.372 (3)
C2—C3	1.373 (2)	C18—H18A	0.9300
C2—H2A	0.9300	C19—C20	1.377 (2)
C3—C4	1.369 (2)	C19—H19A	0.9300
C3—H3B	0.9300	C20—C21	1.386 (2)
C4—C5	1.370 (2)	C20—H20A	0.9300
C4—H4A	0.9300	C21—C22	1.4994 (19)
C5—C6	1.391 (2)	C22—C23	1.5008 (19)
C5—H5A	0.9300	C22—H22A	0.9700
C6—C7	1.500 (2)	C22—H22B	0.9700
C7—C8	1.500 (2)	C23—C24	1.3858 (19)
C7—H7A	0.9700	C23—C28	1.396 (2)
C7—H7B	0.9700	C24—C25	1.374 (2)
C8—C9	1.387 (2)	C24—H24A	0.9300
C8—C13	1.3980 (19)	C25—C26	1.374 (3)
C9—C10	1.375 (3)	C25—H25A	0.9300
C9—H9A	0.9300	C26—C27	1.377 (2)
C10—C11	1.369 (3)	C26—H26A	0.9300
C10—H10A	0.9300	C27—C28	1.3927 (19)
C11—C12	1.373 (2)	C27—H27A	0.9300
C11—H11A	0.9300	C28—C29	1.4735 (18)
C12—C13	1.393 (2)	C29—C30	1.4642 (19)
			
Cg1···Cg1^i^	3.673 (4)	Cg2···Cg2^ii^	3.793 (4)
			
C14—N1—C1	123.45 (11)	N2—C15—C14	175.67 (16)
C29—N3—C16	123.40 (10)	C17—C16—C21	119.88 (12)
C2—C1—C6	119.79 (12)	C17—C16—N3	115.52 (12)
C2—C1—N1	115.43 (11)	C21—C16—N3	124.48 (12)
C6—C1—N1	124.70 (12)	C18—C17—C16	120.68 (14)
C3—C2—C1	120.61 (14)	C18—C17—H17A	119.7
C3—C2—H2A	119.7	C16—C17—H17A	119.7
C1—C2—H2A	119.7	C17—C18—C19	119.75 (15)
C4—C3—C2	119.76 (15)	C17—C18—H18A	120.1
C4—C3—H3B	120.1	C19—C18—H18A	120.1
C2—C3—H3B	120.1	C18—C19—C20	120.00 (15)
C3—C4—C5	120.23 (14)	C18—C19—H19A	120.0
C3—C4—H4A	119.9	C20—C19—H19A	120.0
C5—C4—H4A	119.9	C19—C20—C21	121.66 (15)
C4—C5—C6	121.38 (14)	C19—C20—H20A	119.2
C4—C5—H5A	119.3	C21—C20—H20A	119.2
C6—C5—H5A	119.3	C20—C21—C16	118.00 (13)
C5—C6—C1	118.08 (14)	C20—C21—C22	122.27 (13)
C5—C6—C7	122.46 (13)	C16—C21—C22	119.73 (12)
C1—C6—C7	119.45 (12)	C21—C22—C23	109.89 (11)
C8—C7—C6	110.19 (11)	C21—C22—H22A	109.7
C8—C7—H7A	109.6	C23—C22—H22A	109.7
C6—C7—H7A	109.6	C21—C22—H22B	109.7
C8—C7—H7B	109.6	C23—C22—H22B	109.7
C6—C7—H7B	109.6	H22A—C22—H22B	108.2
H7A—C7—H7B	108.1	C24—C23—C28	118.32 (13)
C9—C8—C13	118.34 (15)	C24—C23—C22	122.21 (13)
C9—C8—C7	122.16 (14)	C28—C23—C22	119.47 (12)
C13—C8—C7	119.49 (12)	C25—C24—C23	120.81 (16)
C10—C9—C8	120.89 (16)	C25—C24—H24A	119.6
C10—C9—H9A	119.6	C23—C24—H24A	119.6
C8—C9—H9A	119.6	C26—C25—C24	120.87 (14)
C11—C10—C9	120.64 (15)	C26—C25—H25A	119.6
C11—C10—H10A	119.7	C24—C25—H25A	119.6
C9—C10—H10A	119.7	C25—C26—C27	119.47 (15)
C10—C11—C12	119.79 (16)	C25—C26—H26A	120.3
C10—C11—H11A	120.1	C27—C26—H26A	120.3
C12—C11—H11A	120.1	C26—C27—C28	120.16 (15)
C11—C12—C13	120.34 (15)	C26—C27—H27A	119.9
C11—C12—H12A	119.8	C28—C27—H27A	119.9
C13—C12—H12A	119.8	C27—C28—C23	120.32 (12)
C12—C13—C8	119.93 (13)	C27—C28—C29	119.32 (12)
C12—C13—C14	119.64 (12)	C23—C28—C29	120.34 (11)
C8—C13—C14	120.42 (12)	N3—C29—C30	113.12 (11)
N1—C14—C13	131.09 (12)	N3—C29—C28	130.40 (12)
N1—C14—C15	113.18 (11)	C30—C29—C28	116.35 (11)
C13—C14—C15	115.56 (11)	N4—C30—C29	178.61 (15)
			
C14—N1—C1—C2	−144.10 (13)	C29—N3—C16—C17	144.15 (12)
C14—N1—C1—C6	39.14 (19)	C29—N3—C16—C21	−39.94 (18)
C6—C1—C2—C3	−4.5 (2)	C21—C16—C17—C18	2.36 (19)
N1—C1—C2—C3	178.60 (12)	N3—C16—C17—C18	178.47 (12)
C1—C2—C3—C4	2.7 (2)	C16—C17—C18—C19	−2.1 (2)
C2—C3—C4—C5	0.7 (3)	C17—C18—C19—C20	0.7 (2)
C3—C4—C5—C6	−2.2 (3)	C18—C19—C20—C21	0.4 (3)
C4—C5—C6—C1	0.4 (2)	C19—C20—C21—C16	−0.2 (2)
C4—C5—C6—C7	179.82 (14)	C19—C20—C21—C22	−179.33 (14)
C2—C1—C6—C5	2.86 (19)	C17—C16—C21—C20	−1.19 (18)
N1—C1—C6—C5	179.49 (12)	N3—C16—C21—C20	−176.93 (12)
C2—C1—C6—C7	−176.53 (12)	C17—C16—C21—C22	177.98 (12)
N1—C1—C6—C7	0.1 (2)	N3—C16—C21—C22	2.23 (18)
C5—C6—C7—C8	114.56 (15)	C20—C21—C22—C23	−116.19 (15)
C1—C6—C7—C8	−66.07 (17)	C16—C21—C22—C23	64.68 (15)
C6—C7—C8—C9	−113.97 (14)	C21—C22—C23—C24	113.44 (15)
C6—C7—C8—C13	66.57 (16)	C21—C22—C23—C28	−66.68 (15)
C13—C8—C9—C10	1.2 (2)	C28—C23—C24—C25	−0.7 (2)
C7—C8—C9—C10	−178.22 (14)	C22—C23—C24—C25	179.17 (14)
C8—C9—C10—C11	0.7 (2)	C23—C24—C25—C26	−0.6 (2)
C9—C10—C11—C12	−1.1 (2)	C24—C25—C26—C27	0.2 (2)
C10—C11—C12—C13	−0.5 (2)	C25—C26—C27—C28	1.4 (2)
C11—C12—C13—C8	2.5 (2)	C26—C27—C28—C23	−2.72 (19)
C11—C12—C13—C14	−176.18 (12)	C26—C27—C28—C29	175.55 (12)
C9—C8—C13—C12	−2.84 (19)	C24—C23—C28—C27	2.32 (19)
C7—C8—C13—C12	176.64 (12)	C22—C23—C28—C27	−177.56 (12)
C9—C8—C13—C14	175.83 (12)	C24—C23—C28—C29	−175.94 (12)
C7—C8—C13—C14	−4.68 (18)	C22—C23—C28—C29	4.19 (18)
C1—N1—C14—C13	0.1 (2)	C16—N3—C29—C30	−177.84 (11)
C1—N1—C14—C15	175.05 (11)	C16—N3—C29—C28	−2.2 (2)
C12—C13—C14—N1	142.00 (14)	C27—C28—C29—N3	−139.03 (14)
C8—C13—C14—N1	−36.7 (2)	C23—C28—C29—N3	39.2 (2)
C12—C13—C14—C15	−32.90 (17)	C27—C28—C29—C30	36.50 (17)
C8—C13—C14—C15	148.42 (12)	C23—C28—C29—C30	−145.22 (12)

Symmetry codes: (i) −*x*+2, −*y*+1, −*z*+1; (ii) −*x*+1, −*y*+1, −*z*.
